# Mr. Evans's Case of Dislocation

**Published:** 1801-06

**Authors:** J. Evans

**Affiliations:** Ketley


### *To the Editors of the Medical and Phyſical Journal.*

GENTLEMEN,

I Have taken the liberty of communicating to you a caſe of diſlocated ſhoulder, ſuch as, I believe, very ſeldom happens; if you think it merits a place in your uſeful Publication, you will oblige me by inſerting it. I am, &c.

*Ketley, April* 18, 1801. J. EVANS.

---

January 9, 1801, Wm. Whittingham, a labouring man, 64 years of age, applied to me with his left ſhoulder apparently much bruiſed, in conſequence of a fall he had met with a few days before. The man was in liquor when the accident happened, ſo that he could give me no ſatisfactory account in what poſition his arm was in at the time he received the injury. On examination, I found the whole ſhoulder ſo much ſwelled that I could not feel either the head of the os humeri or the extremity of the ſcapula; and, as the arm could be brought cloſe to the ſide without difficulty or pain, I of courſe concluded there was no diſlocation; therefore directed the ſhoulder to be well rubbed twice a day with a volatile embrocation, and a ſtale beer poultice to be applied till the tumefaction ſubſided.

In nine days after (about a fortnight from the time of the accident) he came to me again, with his arm and hand much ſwelled, but the ſhoulder greatly reduced in ſize, complaining of conſiderable pain in that part of the arm where the deltoid muſcle is inſerted into the os humeri.

I had now a fair opportunity of examining the true ſtate of the ſhoulder, which, to my great ſurpriſe, I diſcovered to be completely diſlocated *backwards*; the head of the os humeri lying evidently *upon* the ſcapula. In the afternoon of the day he applied to me, I, with proper aſſiſtance, attempted the reduction by making a gradual extenſion with the fore-arm bent, (the method I have adopted with ſucceſs for many years, when the head of the bone lay in the axilla, or under the pectoral muſcle); notwithſtanding repeated trials I gained no advantage. Thus foiled in my efforts, and the patient being a good deal fatigued, I poſtponed any farther attempts until the following morning. In the interim, I recollected Mr. White's (of Mancheſter) method of reducing diſlocated ſhoulders of ſeveral month's ſtanding,* which determined me to adopt the ſame mode of practice, eſpecially as my patient's caſe correſponded much with that of James Dawſon's, mentioned in page 100 of Mr. White's book, excepting that the head of the os humeri was forced *upon* the ſcapula, and not *under* it, as related by Mr. White.

The man being placed in a ſitting poſture on the floor, was kept firmly in that ſituation by an aſſiſtant. Two ſtrong perſons ſtood upon a table, and endeavoured to raiſe the patient by his diſlocated arm, while I attempted to guide the head of the bone into the ſocket, but all our exertions proved ineffectual; which I attributed, in ſome degree, to want of ſufficient power, and alſo to the oppoſition the biceps muſcle (being on the ſtretch) might probably give us. I therefore procured a ſet of braſs pullies, uſually employed for raiſing of timber and other heavy materials, and faſtened them to a ſtrong hook which was fixed in a beam at the top of the room. I then defended the man's arm above the elbow with a thick cotton handkerchief wrapped

* See Caſes in Surgery, page 95.

wrapped ſeveral times around it, over which was applied a ſtrong towel, ſecured firmly to a cord that paſſed over the pullies. One of the aſſiſtants made a gradual and powerful extenſion by pulling at the chord, the other * kept the fore-arm bent, with the elbow in an oblique direction backwards, while I, at the ſame time, with one hand preſſed down the ſcapula, and with the other endeavoured to diſlodge the head of the bone. In the ſpace of a few ſeconds I had the ſatisfaction of feeling it give way, and hearing it crack as it went into its place. Upon which the patient with great extacy cried out, "It was in." He was then ſet at liberty; and, on examination, I found he was perfectly right in his aſſertion. He became immediately eaſier, and by carrying his arm in a ſling for a few days the ſwelling gradually ſubſided. At the end of a fortnight he was able to return to his uſual employ.

